# Medicinal plants and foods with metaphorical concepts in Rumi’s “*Masnavi Manavi*”: The psychosomatic approach to human health

**DOI:** 10.22038/AJP.2023.71710.3309

**Published:** 2023

**Affiliations:** 1 *Department of Persian Language and Literature, Faculty of Literature and Humanity, Ferdowsi University of Mashhad, Mashhad, Iran*; 2 *Department of Physiology, Faculty of Medicine, Mashhad University of Medical Sciences, Mashhad, Iran*; 3 *Division of Neurocognitive Sciences, Psychiatry and Behavioral Sciences Research Center, Mashhad University of Medical Sciences, Mashhad, Iran*; 4 *Applied Biomedical Research Center, Mashhad University of Medical Sciences, Mashhad, Iran*

**Keywords:** Rumi, Masnavi Manavi, Medicinal plant, Medicinal foods, Persian medicine, Spiritual health

## Abstract

**Objective:**

“*Masnavi Manavi*” is one of the most valuable texts of Persian literature. In this book, Rumi (Mevlana) with a unique method and in the form of moral stories teaches life lessons, mystical truths and even therapeutic advices to people. The aim of this study is to highlight the medicinal plants and foods that had been applied both in somatic and spiritual concept in “*Masnavi Manavi*’ poems.

**Materials and Methods:**

For this purpose, a library-based, descriptive and analytical method was used.

**Results:**

Some medicinal plants and food terms such as rose-water, vinegar, honey, oxymel, common reed, grape, onion, garlic and wheat are mentioned in this study to show Rumi's metaphorical and therapeutic approach as a doctor who treats both soul and body. In fact, Rumi's intention to apply these terms was to express his ideas and views about the inseparability of physical and spiritual aspects in human health and well-being.

**Conclusion:**

Rumi focus in “*Masnavi Manavi*” moral stories is the soul health and consider body as carrier of the soul. Therefore, because of this psychosomatic approach to human disease, he selects the most suitable herbs and foods for explaining spiritual and somatic medicine.

## Introduction

In recent decades, experimental and clinical studies have approved the attitude of traditional medicine to the role of spiritual factors in healing and human health (Singh and Ajinkya, 2012 ▶; Tabei et al., 2016 ▶). According to traditional literature, Iranian philosophers and “*hakims”* had a psychosomatic approach to human health even in their non-medical books. Mevlana Jalal-ad-Dın Muḥammad Balkhī known as Rumi is one of the most famous poets and sages of the Persian language, whose book “*Masnavi Manavi*” is known all over the world. “*Masnavi Manavi*” is not only a literary masterpiece but also a prominent work in Islamic mysticism that pays attention to both the physical and spiritual aspects of man (Tabatabaee et al., 2022 ▶). Although Rumi was not a physician, the medicinal terms and concepts in “*Masnavi Manavi*” shows his deep knowledge of the Persian Medicine. Rumi’s knowledge of medicine and his experience of self-medication and use of different remedies during illness helped him to write poems with medical themes such as different kinds of human temperaments and their relationship with diseases, symptoms of diseases and healing methods. He advises common treatments for fevers, gastrointestinal diseases, and neurological and psychosomatic disorders such as disease of love in his poems (Rezaei and Aliakbari, 2013 ▶). 

The theme of many stories in “*Masnavi Manavi*” is about both spiritual and physical illness. Some of them are as follows: “the king's falling in love with a handmaiden” (1:36-246), “begging of the Jesus’s companion” (2:141-155,457-500), “An old man complained to his physician that he suffered from headache” (2:3088-3115), “How the boys made the teacher imagine (that he (the teacher) was ill)” (3:1546-1609), “How Pharaoh was made (spiritually) ill by vain imagination arising from the people's reverence (for him)” (3:1555-1561), “the Sadr-i Jahan's Wakill (minister) ”(3:3686-4810), “the woman whose children never lived (long)” (3:3399-3416), “the tanner who fainted and sickened on smelling otto and musk”(4:257-305), and “the druggist whose balance-weight was clay for washing the head”(4:625-652) (Rumi and Nicholson, 2001 ▶). According to these stories, Rumi had been aware of the complex relationships of the body control system such as the endocrine glands, and the nervous system with the soul and the heart. For example, Rumi has pointed to baby crying and milk ejection from the breasts of mother which is a new finding of modern medicine (Rumi and Nicholson, 2001 ▶). 

"*Until the tender-throated babe is born, how should the milk for it begin to flow from the (mother's) breast?*")3:3213(

He also believe that positivism and happiness is necessary for body health and fitness, while stress and anxious thoughts might result in emaciation and physical weakness (Rumi and Nicholson, 2001 ▶). 

"*Man has fatness from (thrives on) fancy, if his fancies are beautiful*" )2:594(

*"For (mere) hope makes him lean, pale, and wretched: he is not ill with bodily ailment*")5:3629(

He used several medicinal words, idioms and motifs in his poems, including names of diseases, descriptions of doctors (“*tabib”*), medicinal drugs, plants and foods, and different temperaments (Saadati and Mohammadi, 2019 ▶). Rumi divides medical doctors into physician of body who pay attention to symptoms and signs such as pulse and hear rate, urine and nutrient regimen, and physician of soul who treat patients upon the light flushes from the God almighty and describes each one very precisely (Maulana and Hashempour Sobhani, 2002 ▶). So, Rumi concentrated upon his medical knowledge and medical terms including medicinal plants and foods to describe their medical role in the treatment of psychosomatic diseases in his poems.

The aim of this study is to study about the moral lesson and spiritual application of medicinal foods and plant terms in “*Masnavi Manavi*” poems with the help of new evidence obtained from experimental studies. Our approach is neither to focus on medical history nor to show the historical value of medicine. We tried to show dual (somatic and spiritual) use and concepts of some medicinal foods and plants like rose-water, vinegar, honey, oxymel, common reed, grape, onion, garlic and wheat in the verses of “*Masnavi Manavi*”. 

## Materials and Methods

In this study, a library-based, descriptive and analytical method has been used. The index of verses of the “*Masnavi Manavi*” index book (in Persian, compiled and edited by Tofiq Hashempour Sobhani (Maulana and Hashempour Sobhani, 2002 ▶)) was searched to find the names of medicinal foods and plants in the verses. The index of verses containing all verses in two parts (the first hemistich and the second one), so it is a complete list of poems keywords. For this study both two parts (hemistich) were thoroughly examined for finding the medicinal plants and foods. After studying the related verse, the medicinal plants and food which have been applied both in somatic and spiritual concepts have been included in this study. Some medicinal plants and food terms such as rose-water, vinegar, honey, oxymel, common reed, grape, onion, garlic and wheat have been chosen and their related verses were mentioned in this study as samples to show Rumi's metaphoric and therapeutic approach. English translation of “*Masnavi Manavi*” by Nicholson was used to mention the related poems, and verses were highlighted by italic font (Rumi and Nicholson, 2001 ▶). 

**Rose-water**

“*Golab*” or rose-water (which is produced by distilling rose flower petals in steam) is a main product of *Rosa damascene* and in traditional medicine is prescribed as heart tonic, quencher of body temperature and thirst, and to relief tension and grief (Boskabady et al., 2011 ▶). *Rosa damascene* and other plants from the Rosacea family are widely used in perfume, cosmetic and food industry (Mileva et al., 2021 ▶). In south Khorasan, Iran, rose-water is made from a type of rose called fire-flower which, like *Rosa damascene*, flowers only in spring (Ekhtiari, 2015 ▶). Rose oil as a product of rose-water is used also in perfume industry. The main components of rose oil show several activities such as: anti-inflammatory, anti-oxidant, anti-diabetic, lipid lowering, anti-convulsant and anti-epileptic functions. Rose oil is also used as a preservative, antibacterial, antifungal, anti-hypertensive and anticancer agent (Mileva et al., 2021 ▶). Moreover, the topical administration or aromatherapy with rose oil or rose-water had been shown to have analgesic, and psychological and physiological relaxing effects (Mohebitabar et al., 2017 ▶; Barati et al., 2016 ▶).

The word rose-water has been mentioned in several verses of the “*Masnavi Manavi*”*.* In some of them, Rumi has pointed out the healing properties of rose-water, which was able to bring an anesthetized person to consciousness. For example, in the following two verses, he mentions these effects (Rumi and Nicholson, 2001 ▶). 

“*they were sprinkling rose-water on his head and face; they were unaware of the rose-water of his love*” (3:3868) 

“*whatsoever they applied of incense and rose-water, he neither stirred not spoke*” (3:4618)

In the story of the Sadr-i Jahán's Wakíl (minister), rose-water was not effective as a herbal remedy because Wakíl had a love disease and Rumi used this word to refer to another concept and meaning, which is real love. While in another story, prescribing the rose-water showed healing effect (Rumi and Nicholson, 2001 ▶). 

“*they sprinkled water and rose-water on the face of the Súfí, that he might recover from his unconsciousness and the sleep (of his senses); when he came to himself, he saw the party (of soldiers), and they asked him how it had happened*” (Rumi and Nicholson, 2001 ▶). 

This dual approach and the importance of paying attention to appropriateness of treatment with lifestyle and mental state was also emphasized in the following verses. In the following verses the importance of matching medicine with patient situation was suggested (Rumi and Nicholson, 2001 ▶).

“*if the beetle feels a desire for some (particular) rose-water, that constitutes a proof of its not being rose-water*” (2:2086) 

“*one was putting his hand on his (the tanner's) heart, while another sprinkled rose-water upon him*” (4:261)

 “*he did not know that from (smelling) rose-water in the meadow (the bazaar) that calamity had overtaken him*”(4:262) 

“*He (the tanner), from carrying dung, has become like the dung-beetle: the dung-beetle is made insensible by rose-water*” (4:278) 

“*the sincere mentors prepare medicine for him (the wicked man) with ambergris or rose-water to open the door (of Divine Mercy)*” (4:281)

If the medical doctor did not pay attention to the patient’s situation, his prescription might worsen the situation and have the opposite effect. In these stories, the beetle and dung-beetle are used to the bad smell of dung for long time, so rose-water had the opposite effect on them. In other verses, Rumi uses rose and rose-water as a medicine for the soul, and in the sense of a prophet and informative matter (Rumi and Nicholson, 2001 ▶). 

“*Since the rose is past and the garden ravaged, from whom shall we get the perfume of the rose? From rose-water*” (1: 672)

“*By means of rose-water and (other) remedies he came to himself (again): little by little, good and evil were apprehended by him (once more)*” (4:3184)

"*if there be any musk or rose-water, I will smell it: this is my art and science and knowledge*” (4:2392)

 “*If your thought is a rose, you are a rose garden, and if it is a thorn, you are fuel for the bath-stove; If you are rose-water, you are sprinkled on head and bosom, and if you are (stinking) like urine, you are cast out; Look at the trays in front of druggist each kind put beside its own kind*” (2: 278-280)

**Vinegar, honey and oxymel**

Vinegar is historically considered a natural remedy and functional food because of many health promoting and medicinal properties (Samad et al., 2016 ▶). Vinegar, especially the old one, is effective in the regulation of serum glucose, lipid disorders, and allergic disease. Moreover, the antioxidant, anti-inflammatory, and anti-microbial effects of vinegar have also been reported (Ousaaid et al., 2021 ▶). A literature review from 1995-2018 reported that daily intake of 10-30 ml of vinegar resulted in a good glycemic response in people with carbohydrate rich regimen (Santos et al., 2019 ▶). In another study, 60 type 2 diabetic patients were divided into control and case group which received 15 ml apple cider vinegar for one month .They reported that fasting blood sugar reduced from 175 to 156 mg/dl and hemoglobinA1c from 7.56 to 7.03 % in case group (Mahmoodi et al., 2013 ▶). Daily intake of 0.5 % vinegar in a regimen for 72 days prolonged the *tumor*-bearing mice (with sarcoma 180 cell line and colon 38 cell line implementation) life span, which was attributed to antioxidant and reactive oxygen scavenging of the fruit vinegar (Seki et al., 2004 ▶). Fermented vinegar is one of the two main ingredients in the production of a medicinal drink known as “*sekanjabin*” in Persian and oxymel in Latin. World famous physicians (*Hakims*) such as Galen, and Avicenna used oxymel for different disorders such as pain, asthma, cough, dyspnea and cold. Rumi in “*Masnavi Manavi*” describes oxymel as a remedy for thirst and as a foodstuff; he also describes oxymel as a bile reducing which is suitable for bilious temperament (Orhan et al., 2022 ▶). In a recent clinical study, consumption of 30 ml oxymel in obese and overweight peoples (both sexes) for 30 days, total cholesterol and body weight significantly decreased in comparison with control group; although it had no effect on other parameters (Marjan et al., 2021). In Persian traditional medicine, several type of “*sekanjabin*” were made by mixing and boiling different proportion of honey (or sometimes sugar) and vinegar. Based on pharmaceutical literatures each kind of “*sekanjabin*” had been prescribed and suitable for a special type of temperament and disease. In “*sakanjbin*”, the cold and sour taste of vinegar is modified by the warm and sweet taste of honey (Zargaran et al., 2012 ▶). In addition, recent studies indicated the presence of dopamine, melatonin and serotonin in honey. These biological regulators are among the most important neurotransmitters in mind and spiritual health (Kim et al., 2022 ▶). Considering this fact, Rumi used the words vinegar, honey and “*sakanjbin*” in both their close meaning and medical application, as well as in esoteric and mystical meaning (Orhan et al., 2022 ▶). In the sixth Book of “*Masnavi Manavi*”, Rumi has likened the calling of the prophets in this world to giving thanks (sweetness like honey) and the sourness of the opponents of the prophets to pouring vinegar. The more they poured vinegar; the prophets not only did not stop their witty invitations, but also increased their thanksgiving (as much as the vinegar increases acidity, therefore, it is necessary to increase the sugar). The following verses are a description of the stubbornness of Noah's people and his efforts at invitation of those ignorant (Rumi and Nicholson, 2001 ▶).

 “*Wrath is (like) vinegar, mercy like honey; and these twains are the basis of every oxymel*” (6:18) 

“*If the honey fails to withstand (be overpowered by) the vinegar, the oxymel will be spoilt*” (6:19) 

“*The people were pouring vinegar on him (Noah), and the Ocean (of Divine Bounty) was pouring more sugar for Noah*” (6:19) 

In addition, following verses refer to psychosomatic and balancing effect of vinegar and honey combination (Rumi and Nicholson, 2001). 

“*I was woven (mingled) together, like honey and vinegar, that I might find the way to (cure) sickness of heart. If vinegar wants (to find) the way to liver, let it becomes oxymel by (being mixed with) sugar*” (1:3663)

 In this verse, Rumi mentions that the prophet had both humanity and spiritual characteristic as a role model to help people for reliving spiritual diseases. In the next verse, vinegar is a symbol of human trait like anger and honey is a symbol of spiritual qualities like compassion and love (Rumi and Nicholson, 2001 ▶). 

“*Since thou hast recovered from thine illness, O thou (that wert) in thrall (to it), leave the vinegar and continue to eat the honey*” (1:3664) 

The above-mentioned verses and the following show that Rumi knew that in traditional medicine, simple “*sakanjbin*” is suitable for most temperaments, especially a bilious temperament and disease related to an increase in body warmth (Afshari M et al., 2011 ▶). As in the story of king and handmaiden, the physicians use medication such as “*sakanjbin*”, almond and myrobalan to restore her health by decreasing bile and constipation (Rumi and Nicholson, 2001). 

“*By Divine destiny, oxymel produced the bile, and oil of almonds was increasing the dryness*” (1:54) 

“*From (giving) myrobalan constipation resulted, relaxation ceased; and water fed the flames, like naphtha*” (1:53) 

However, those medications were not effective, because the handmaiden suffered from love sickness, which in modern medicine is known as anorexia nervosa (Nimrouzi and Zarshenas, 2018 ▶). In this story, Rumi warns that the medical doctor should pay attention to the psychological aspects of the patient because the wrong treatment might threaten the patient's life. 

**Reed and mithridate (** ***Phragmites australis*** **)**

*Phragmites australis* or common reed (“*nay”* in Persian) has many medicinal properties such as antiviral, antioxidant, and anti-melanogenesis effects and used to treat diarrhea, fevers, vomiting, lung abscesses, coughs with thick dark phlegm, urinary tract infections and livestock illnesses in both traditional and experimental pharmacology (Sohaib et al., 2022 ▶; Zhu et al., 2017 ▶). In a study, 21 endophytes bacteria were isolated from *Phragmites australis *and examined against 36 samples of (multi-drugs) resistant pathogens isolated from clinical patients, hospitals and food. Seven out of 21 endophytes bacteria isolated from the “*nay”* belonged to *Pseudomonas*, and *Stenotrophomonas* family and were able to inhibit the growth of most of the pathogens isolated from foods (Delfino et al., 2021 ▶). There is a type of reed with a sweet taste which is named sugar cane. Rumi applied this word to describe spiritual pleasure and sweetness of soul and heart during spiritual experience, and use it as a metaphor to refer to the Sufi and saints of God (Rumi and Nicholson, 2001 ▶). 

“*Both reeds drank from the same water-source, (but) this one is empty and that one full of sugar*” (1:270) 

“*Whether in the earth there are sugar-canes or only (common) reeds, every earth (soil) is interpreted by its plants*” (4:1317)

In these verses, Rumi explained the difference between superficial people and spiritual saints. Although both of them are human with similar instinctive needs, however in saints inside, drinking water becomes Divine light and love (Rumi and Nicholson, 2001 ▶).

 “*You give the enemy wine and sugar-cane—for what reason? Bid him laugh venomously and eat earth*” (5:3492)

In the above verse sugar-cane means fulfilling the ego desire.

“*No other blessing will be desired (by me): I will not be diverted from this (delight) by the houris and sugar-cane (of Paradise)*” (4:687)

“*Mind gains strength from another mind: the sugar-cane is made perfect by the sugar-cane*” (2:2277)

“*I am a mine of candy, I am a plantation of sugar-canes: it is growing from me, and at the same time I am eating (of it)*” (2:2428)

In the above verses, sugar-canes is a metaphor for spiritual knowledge and wisdom (Rumi and Nicholson, 2001 ▶). Therefore, “*nay”* in “*Masnavi Manavi*” is a code (clue) for human who was kicked out of heaven and now is eagerly awaiting to make a way to his Lord. In other words, “*nay”* is an instrument that spiritually connects human to almighty God (Ekhtiari, 2002 ▶). 

However, there is a type of poison reed with the scientific name of *Juncus inflexus *and the Latin name of hard rush. This reed is toxic for all types of animals and induces gastric irritation, diarrhea, blindness and rage in poisoned animal (Mirheydar, 1996 ▶). The most important and opening verses of “*Masnavi Manavi*” is “*Nay-Naame*” which expresses the aim of the whole book. Rumi used the word reed (“*nay”*) as a metaphor and asks about the mysterious effect of “*nay”* which might induce dual effect like pain (poison) and/or cure (antidote) (Rumi and Nicholson, 2001 ▶).

“*Who ever saw a poison and antidote like the reed? Who ever saw a sympathiser and a longing lover like the reed?*” (1:12) 

This plant has also had been used as musical instruments like flute and its music can resonate natural rhythm in our body and induce balance between body, brain and soul (Laksmidewi et al., 2019 ▶). The following verses refer to therapeutic effect of reed as musical instruments (Rumi and Nicholson, 2001 ▶).

 “*The reed tells of the Way full of blood and recounts stories of the passion of Majnún*” (1:13) 

“*If the reed had no converse with his lip, the reed would not fill the world with (music sweet as) sugar*” (6:2006)

Moreover, Hafiz, a famous Iranian poet, described reed both as a sugar-cane and as a musical instrument. The following verse in poem 471, he says why people do not pay a sugar-cane to one who gives spiritual sweetness by flowing reed (Bicknell, 2012 ▶).

“For one reed of his candy, why purchase they not that one ***Who, a hundred sugar-scatterings, made from the reed of a single pen” 

**Ripped and unripe grape**

Grape (*Vitis vinifera*) and unripe grape are considered a natural remedy in traditional and modern medicine because of high content of carotenoids and polyphenolic antioxidant. In Persian medicine and Iranian culture, unripe grape (“*Ghura”*) and its juice (verjuice) had been widely used in food and for controlling hyperlipidemia and hypertension (Zolfaghari et al., 2015 ▶). In addition, both experimental and clinical studies demonstrated several pharmacological effects for *Vitis vinifera*. Skin protection, anti-inflammatory, antibacterial, anticancer, antidiabetic, and antioxidant activities, as well as neuroprotective, cardioprotective, and hepatoprotective properties are the pharmacological effects which have been shown for *Vitis vinifera *(Nassiri-Asl and Hosseinzadeh, 2016 ▶). In the following verse, Rumi mentioned the cold and dry temperament of unripe grape and the process of ripening would result in ripped grape and then currant (raisins) which have hot and humid temperament in nature. 

"*In the young grape (“ghura”) the juice is sour, but it is sweet and good when the ghúra comes to be an angúr (ripe grape)*" (1:2601) 

"*Notwithstanding such an incapacity and remoteness (from God), will He confer on these unripe grapes (“ghura”) of mine a perfection like that of the ripe grape (angúr)?*" (6:4740) 

In those verses Rumi used “*Ghura”* as a concept for unexperienced and raw person that is violent and nervous in manners and after going through the stages of mystical behavior became ripped and sweet like grape and full of patience and peace in temper. The process of maturation of the seeker of the right path had been mention in the next verse (Rumi and Nicholson, 2001 ▶). 

“*Thou wast fire: thou hast become light, O noble one; thou wast an unripe grape: thou hast become a (ripe) grape and raisin”* (4:3421) 

However, Rumi believes that education is not effective for some people and training of the incompetent person is like those round objects on the dome (Rumi and Nicholson, 2001 ▶).

 “*If fruit become old, (yet) so long as it is immature and not ripe it is called ghúra (unripe grapes*)” (6:4736) 

“*Though (one resembling) immature and sour (fruit) reach the age of a hundred years, he is (still) a child and unripe (ghúra) in the opinion of every sagacious person*” (6:4737)

**Onion and garlic**

Many medicinal properties including antihypertensive, anti-hyperlipidemic, anti-obesity, anti-inflammatory, anti-oxidant, and antidiabetic effects have been attributed to *Allium* genus vegetables (onion and garlic)(Asemani et al., 2019 ▶; Fakhar et al., 2017 ▶). Both of these vegetables have pungent and unpleasant smell because of various sulfur compounds (Asemani et al., 2019 ▶). A meta-analysis of the reports of 14 studies (published between 1981-2016) showed that intake of garlic has antihyperlipidemic activity because it reduced the level of total cholesterol and low-density lipoprotein instead of triglyceride and high-density lipoprotein (Sun et al., 2018 ▶). Based on epidemiological studies *Allium* vegetable (garlic and onion) consumption is also reported to reduce the risks of different kinds of gastrointestinal cancers such as gastric and colorectal cancers (Nicastro et al., 2015 ▶). In “*Masnavi Manavi*” the word onion and garlic had been used as metaphor for the concept of unpleasant mood such as proudness, avarice and greed not as food (Rumi and Nicholson, 2001 ▶). 

"*The smell of pride and the smell of greed and the smell of concupiscence will become, in speaking, like (the smell of) onions*" (3:166)

"*If thou take oath, saying, “When have I eaten them? I have abstained from onions and garlic*” (3:167) 

The presence of these vicious traits in a person likened to a bad smell which come from his interior and his act or talking show his inside mood and selfish trait. In the next verse the word onion, leek, and poppy are as metaphor for inner secrets of humanity which will be revealed on the Day of Judgment. From onion, leek, and poppy the hand of spring reveals the secret of winter (Rumi and Nicholson, 2001 ▶). 

"*As the spring season comes, plants grow and it becomes clear what plants the earth has hidden in its heart*"(5:1801)

In the story of how that seeker was content to be taught the language of domestic fowls and dogs, and how Moses, on whom be peace, complied with his request. Rumi explains the reason of that desire which is that a person does not give up materialistic attachments as long as he has not achieved higher values. In next verse Rumi gave an example which means that until a child sees an apple, he will not miss the smelly onion (Rumi and Nicholson, 2001 ▶). 

“*Till a child sees that the apple is there, it will not give up from its hand the stinking onion*’’ (3: 3355) 

**Wheat**

Wheat is a major source of energy and contain several compound including protein, dietary fiber, phytochemicals and vitamins (notably B vitamins) which are essential or effective for human health (Shewry and Hey, 2015 ▶). Wheat contain several phytochemicals including phenolic acids, flavonoids, tocotrienols, tocopherols, carotenoid, alkylresorcinols, phytosterols and benzoxazinoids. Some of these are considered natural antioxidant (Tian et al., 2022 ▶). A meta-analysis study showed a reverse correlation between consumption of whole wheat intake and risk of colorectal cancer (Benisi-Kohansal et al., 2016 ▶). Whole grain because of containing phytochemicals such as alkylresorcinols, phenolic acids, and phytic acid has an important role in diabetes prevention by contributing to the control of blood insulin sensitivity, hyperinsulinemia and serum glucose levels (Biskup et al., 2017 ▶). Trans-ferulic acid (FA) is the main form of phenolic acids in wheat. Beneficial effects on obesity and diabetes, reducing risks of cardiovascular diseases and reducing risks of cancers have been reported for FA (Adam et al., 2002 ▶). Rumi applies the word of wheat as a food and nutrient for both body and spirit. In the following verses, Rumi mentions wheat as a blessing material in the process of production and use, as well as a symbol for the stages of human growth and development in spiritual conduct (Rumi and Nicholson, 2001 ▶). 

"*They cast a grain of wheat under earth, then from its earth they raised up ears of corn; Once more they crushed it with the mill: its value increased and it became soul invigorating bread; Again they crushed the bread under their teeth: it became the mind and spirit and understanding of one endowed with reason*" (1:3165-3167)

**Saffron**

*Crocus sativus* or saffron has been cultivated and traditionally used as food additive, dietary spice, and medicinal plants in various countries such as Iran, Turkey, Spain, and Greece. In modern and traditional medicine saffron stigma is suggested as an effective remedy in prevention and treatment of several disease (Hosseinzadeh and Nassiri-Asl, 2013 ▶). Medicinal properties such as anti-depressive, tranquilizer, eupeptic, sedative, antispasmodic, appetizer, expectorant, anti-inflammatory and anticancer have been showed for this functional herb (Khazdair et al., 2015 ▶; Boskabady et al., 2020 ▶; Gholamnezhad et al., 2013 ▶). In addition, anti-anxiety and anticonvulsant properties of safranal (responsible for the aroma and odor of saffron) had been shown in preclinical studies which suggested it as a potential aromatherapy and hypnotic agent (Rezaee and Hosseinzadeh, 2013 ▶). 

In Persian medicine the term saffron refers to a medicinal plant which especially is joyful and sedative, and is a symbol of expressing the sign of disease because of its yellow color. In Persian literature also attention had been paid to joyful and sedative effects of saffron. Sanaei who is a great poet says: 

“*the one who eat saffron would be fresh and attractive, while the one who grind and powder saffron speak falsely and express confused words*” (Sanā'ie, 2018 ▶). 

Another famous Persian poet Hakim Khaghani also noted a conflict between the joyful effect of saffron on heart and the color of saffron, which is yellow and is used to describe sadness and illness in a person (Khaqani Sharvani, 1989 ▶). Rumi with his knowledge of medicine uses saffron as a metaphor for both of these concepts, as a marker of health and happiness or as a symbol of disease, sorrow and fear. In the next verse saffron is a metaphor for happiness, while onion is a metaphor for separation. Because eating saffron makes person laughing and feel cheerful, as similar as the mood of lovers after marriage. The smell of onion irritates the eyes' lachrymal glands and makes person crying as the feeling of a person after separation from a lover (Rumi and Nicholson, 2001 ▶). 

"*The scent of the saffron of union produces (happy) laughter; the smell of the onion of absence (produces) tears*" (6:4042) 

In the following verses, saffron is a symbol for extreme fear. Rumi also used saffron as metaphor for the joy of intuition and common sense (Rumi and Nicholson, 2001 ▶).

*''There was a decrepit old woman aged ninety years, her face covered with wrinkles and her complexion (yellow as) saffron*'' (6:1222) 

"*The colour of red anemone becomes the colour of saffron; his lion-like strength becomes as the courage of women*'' (5:971)

In the next verse, Rumi tells us that a believer and a seeker person should drink the water of lord obey and worship in the field of the world in order to reach the pleasure of discovering the facts and intuition. (Rumi and Nicholson, 2001 ▶).

"*Drink the water, O saffron, that thou mayst attain to maturity: thou art saffron, thou wilt attain to that halwá (sweetmeat)*" (4:1086)

## Discussion

A multidimensional and holistic approach is one of the important characteristics in the content of poems of Persian poets. Most of the Iranian poets tended to present new and metaphoric literary expression by using scientific terms (Tabatabaee et al., 2022 ▶). The wide presence of these motifs in Rumi poems is a sign of his deep insight into the problems of society and his knowledge of the different sciences of that time (Saadati and Mohammadi, 2019 ▶). 

Medicinal plants and foods were one of the main basis of healing procedures of ancient medicine (Shabab et al., 2021 ▶). The dual concept of medicinal plant and food in “*Masnavi Manavi*”’s poems might demonstrate that the Rumi approach to human could be considered a suitable model to prove the inseparability of the body and the soul. 

Rumi’s attention to both physical and spiritual aspects of human clearly show that he was aware of the inseparability of these. Perhaps this model of Rumi approach to medicine could be a good model for the treatment of human diseases. However, Rumi does not encourage other poets to use metaphoric concepts in their poems. Rumi concentrated upon his medical knowledge and medical terms including medicinal plants and foods to describe their medical role in the treatment of psychosomatic diseases in his poems. Some of the medical foods described by Rumi nowadays are also in use, such as vinegar, “*sekanjabin*”, and *Vitis vinifera*. Rumi believes that medicinal knowledge has been obtained through both experimental studies and divine knowledge (Saadati and Mohammadi, 2019 ▶; Rumi and Nicholson, 2001 ▶). He mentioned the story of Prophet Solomon, who discovered the properties of medicinal herbs from Divine source, which later was experienced and practiced by physicians and mentioned in medicinal book. Rumi says: 

*"Every morning, when Solomon came and made supplication in the Farther Mosque, He saw that a new plant had grown there; then he would say, “Tell thy name* *and use. What medicine art thou? What art thou? What is thy name? To whom art thou hurtful and for whom is thy usefulness?”*

*Then every plant would tell its effect and name, saying, “I am life to that one, and death to this one I am poison to this one, and sugar to that one: this is my name (inscribed) on the Tablet by (the pen of) the Divine decree.”*

*Then (by hearing) from Solomon about those plants, the physicians became learned and wise authorities (on medicine)* (4:1286-1293)

So that they compiled medical books and were relieving the body from pain. This astronomy and medicine is (knowledge given by) Divine inspiration to the prophet: where is the way for intellect and sense (to advance) towards that which is without (spatial) direction" (Rumi and Nicholson, 2001 ▶).

Considering a Divine source for medicine might explain the psychosomatic approach and dual use of therapeutic issues (treatment modalities and herbal remedies) in “*Masnavi Manavi*” poems. Rumi employed some medicinal plants and food terms such as rose-water, vinegar, honey, oxymel, common reed, grape, onion, garlic and wheat have been mentioned to show his metaphoric and therapeutic approach as a doctor of soul and body. This psychosomatic approach had been noticed in modern medicine too. New experimental and clinical evidence of cardiac and cerebral effects of medicinal foods and plants demonstrated the importance of the role of the heart and brain in traditional Persian medicine, and the close interaction of body and mind health. In fact, Rumi's intention to apply these terms was to express his ideas and views about the inseparability of physical and spiritual aspects of human health and well-being. Moreover, Rumi's intention was not to be a narrator or a historian, but he only wants to tell his spiritual and inspirational epics in the form of moral stories, and these anecdotes should be considered as a tool for him to advise methods for healing the human body and soul. Although Rumi is not a medicine practitioner, but like Avicenna's (Ibn Sina) knew the value of love in the individual health and treatment of disease (Ekhtiari, 2009 ▶; Sparham and Tasdighy, 2018 ▶). In most of the “*Masnavi Manavi*” stories, he described and introduced the true love as the main remedy to human pains (Sheikh, 2005 ▶). Therefore, Rumi focus in “*Masnavi Manavi*” moral stories is soul health and he considered the body as a carrier of the soul and because of this psychosomatic approach to disease, he selects the most suitable herbs and foods for explaining both spiritual and somatic medicine. This union approach of Rumi and other Persian-speaking sages and poets to the universe and human being is now be considered as Gestalt Therapy in psychology and life philosophy (Kaya et al., 2014 ▶). In this holistic approach an individual understand and accept his responsibility about her/his needs, fears and desires, and improve her/his self-efficacy which is one of the main factor in management of chronic disease (Hamidi et al., 2022 ▶). 

As limitation, in this study some “Masnavi Manavi” poems mentioning medicinal plants and food terms such as rose-water, vinegar, honey, oxymel, common reed, grape, onion, garlic and wheat are exampled to show Rumi's holistic approach to health.

Rumi focus in “*Masnavi Manavi*” moral stories is the soul health and consider body as carrier of the soul. Therefore, because of this psychosomatic approach to human disease, he selects the most suitable herbs and foods for explaining spiritual and somatic medicine. Rumi approach to human could be considered as a suitable model to prove the inseparability of the body and the soul.

## Conflicts of interest

The authors have declared that there is no conflict of interest.
